# Prevalence and Predictors of Tobacco Use Among Adolescents in Ibadan, Nigeria

**DOI:** 10.5888/pcd20.220234

**Published:** 2023-05-18

**Authors:** Ikenna Onoh, Magbagbeola David Dairo, Muhammad Shakir Balogun, Olufunmilayo Fawole

**Affiliations:** 1 Nigeria Field Epidemiology and Laboratory Training Programme, Abuja, Nigeria; 2 Nigeria Centre for Disease Control, Abuja, Nigeria; 3 Department of Epidemiology and Medical Statistics, Faculty of Public Health, College of Medicine, University of Ibadan, Ibadan, Nigeria; 4 African Field Epidemiology Network, Abuja, Nigeria

## Abstract

**Introduction:**

Most tobacco use begins in adolescence, causing dependence and prolonged use, and accounts for more than 8 million deaths worldwide annually. Monitoring adolescent tobacco use is critical to controlling it. Our study examined the prevalence and factors associated with tobacco use among adolescents in Nigeria.

**Methods:**

We conducted a descriptive cross-sectional study among adolescent students in Ibadan, Nigeria, aged 11 to 18 years, from March through June 2021. We used a 2-stage cluster design to select 3,199 students from 23 schools. We adapted the Global Youth Tobacco Survey Core Questionnaire, version 1.2, for data collection and used logistic regression to assess factors associated with current tobacco use. We weighted all analyses for complex survey design and differential nonresponse at school, class, and student levels.

**Results:**

Prevalence of current use of cigarettes, smokeless tobacco, or any tobacco were 1.4%, 1.1%, and 2.0%, respectively. Predictors of current tobacco use were male sex (adjusted odds ratio [aOR] = 3.13; 95% CI, 1.53–6.42); close friends as smokers (aOR = 3.10; 95% CI, 1.77–5.41); classmates as smokers (aOR = 3.12; 95% CI, 1.15–8.49); access to cigarette (aOR = 6.65; 95% CI, 2.55–17.33); perception that smoking is attractive (aOR = 3.15; 95% CI, 1.17–8.44); exposure to secondhand smoke (aOR = 2.93; 95% CI, 1.07–8.03); and internet awareness of tobacco use (aOR = 3.22; 95% CI, 1.48–7.04).

**Conclusion:**

Prevalence of adolescent tobacco use was low in Ibadan. Predictors were peer influence, access to cigarettes, misperceptions about tobacco use, exposure to secondhand smoke, and tobacco advertising. We recommend an antitobacco campaign that uses a peer education strategy, a comprehensive enforcement of tobacco advertising, and a ban on public smoking.

**Summary**

**What is already known on this topic?**

Most tobacco use starts by adolescence, and among Nigerian children, begins as young as age 7 years. This results in dependence, prolonged use, and resultant health consequences. The current status of adolescent tobacco use and associated factors in Ibadan, Nigeria, is unknown.

**What is added by this report?**

We found a prevalence of adolescent tobacco use in Ibadan of 2%. Adolescent tobacco use was associated with peer influence, cigarette access, misperceptions about tobacco use, exposure to secondhand smoke, and tobacco advertising.

**What are the implications for public health practice?**

Nigeria needs a comprehensive national adolescent tobacco control strategy centered around an antitobacco campaign and enforcement of bans on tobacco advertising and public smoking that is guided by continuous surveillance and monitoring.

## Introduction

Tobacco use is an important preventable cause of premature death and accounts for the deaths of up to half of its users (1). In 2017, about 8 million deaths worldwide were attributable to tobacco, mostly from smoked tobacco (2). If optimal control measures are not implemented, these deaths are projected to reach 10 million by 2030, mostly in low- and middle-income countries (3) where the high burden of tobacco use is due to an ongoing transition from tobacco production to tobacco consumption (4).

Africa has become attractive to multinational tobacco companies because of economic and personal income growth (5), an unsaturated market, weak tobacco-control policies, reduced awareness of the dangers of tobacco use, prioritization of control of infectious disease over noncommunicable diseases, and a young, growing population (4,6,7). Tobacco companies have adapted aggressive and uncontrolled marketing and promotional activities on the continent (6).

Worldwide, nearly all tobacco use begins in childhood and adolescence. Eighty-eight percent of adult daily smokers experiment with their first cigarette by age 18 years (8). Countries with the highest recent increases in tobacco use among their youth are generally lower human development index countries (a summary composite measure of a country's average achievements in 3 basic aspects of human development: health, knowledge, and standard of living) (9). Globally, 43.8 million (12%) of adolescents aged 13 to 15 years use some form of tobacco (10). Adolescents in this age group in low- and middle-income countries have average tobacco use prevalence rates that range from 11% to 13% (10). The younger children are when they start smoking, the less likely they are to quit (11), with prolonged exposure leading to earlier and more pronounced health risks (12). The short-term health consequences of smoking include respiratory effects, such as exacerbation of severe asthma, pneumonia, ear infections, addiction to nicotine, and the risk of other drug use (13,14). In adults, the cumulative effects of tobacco use manifest as cardiovascular diseases, cancers, chronic respiratory diseases, type 2 diabetes, immune and autoimmune disorders, and eye disease (15). Early signs of these diseases occur in adolescents who smoke. Early abdominal aortic atherosclerosis, which affects the flow of blood to vital organs, has been found among young smokers. This leads to consequences such as hypertension, ischemic heart disease, and chronic obstructive pulmonary disease later in life (8).

A primary preventive approach that focuses on pre-adolescence and early adolescence is imperative. In response to the “tobacco epidemic,” the World Health Organization’s (WHO’s) Framework Convention on Tobacco Control (FCTC) (16) was adopted in 2003 and operationalized in 2005. WHO’s FCTC prioritizes the need for an effective surveillance system to monitor tobacco use. The Global Youth Tobacco Survey (GYTS), 1 of 4 surveys conducted within the Global Tobacco Surveillance System, is a school-based survey at a defined geographic site (17). GYTS collects data on students by using a standardized methodology (17). It includes questions on tobacco use and on knowledge and attitudes about tobacco, secondhand smoke (SHS) exposure, tobacco advertising exposure, tobacco cessation, access to tobacco products, and school teaching on tobacco use (18). The survey is repeated every 4 to 5 years, generating data that are comparable within and across countries (17).

Like other sub-Saharan African countries, Nigeria’s young and growing population (19) is attractive to the tobacco industry. Nigeria hosts transnational tobacco companies, including British America Tobacco Nigeria and Japan Tobacco International. Every year, more than 16,100 of Nigeria’s population die from tobacco-caused disease; 748,800 Nigerians aged 15 years or older and more than 25,000 aged 10 to 14 years use tobacco every day (20). Nigeria signed the WHO FCTC in 2004, ratified it in 2005 (21), and domesticated it through the National Tobacco Control Act of 2015 (22).

Two rounds of GYTS (2000 and 2008) were conducted in Nigeria, the last round in 1 state and 4 major cities, including Ibadan. At the time of the surveys, current cigarette use ranged from 2.6% to 6.2%, and current use of other tobacco products ranged from 13.1% to 23.3% (23). No follow-up survey has been done to date, and current levels of tobacco use among Nigerian adolescents is unknown. We addressed this information gap by determining the prevalence of tobacco use and its predictors among school-age adolescents in Ibadan, Nigeria.

## Methods

### Study area

Ibadan, the capital of Oyo state, lies in southwestern Nigeria. Its urban area is divided into 5 local government areas: Ibadan North, Ibadan North East, Ibadan North West, Ibadan South East, and Ibadan South West. With an estimated population of 3,552,000 (24), Ibadan is the third most populous city in Nigeria and is home to several industries, including tobacco processing and cigarette manufacturing, and numerous bars, lounges, and night clubs where smoking is the cultural norm. Several public and private primary and secondary schools are in the city. The secondary school net attendance ratio — the proportion of official school age children participating in schooling — in Oyo State is 66.6% (25).

### Study design and study population

Ours was a school-based, cross-sectional study among adolescents in grades 8 to 10 (Junior Secondary 2 and 3 or Senior Secondary 1), in public or private secondary schools in all 5 local government areas. These grades usually correspond to ages 13 to 15 years, the target age range for GYTS. Students were included in the study if they were in Junior Secondary 2 or 3 or in Senior Secondary 1 in selected schools. All students in selected classes were eligible to participate in the survey. Students in the selected classes who were absent on the day of data collection or who refused to participate were excluded from the survey.

### Sample size and sampling technique

We computed the minimum sample size required for the study by using the formula for estimating a single proportion (26). After applying a cluster design effect factor of 1.5 and a 20% adjustment for nonresponse, the sample size was approximately 1,884 students. This corresponds with the minimum sample size of 1,875 students from 25 schools required for studies using the GYTS methodology (27).

We used a 2-stage cluster sample design. Schools were selected at the first stage by systematic selection with probability proportional to the enrollment size, followed by systematic random selection of classes in selected schools.

The sampling frame for the first stage included all private and public secondary schools in Ibadan containing any of the desired grades obtained from the most up-to-date school enrollment list from the Oyo State Ministry of Education. We listed the schools in the sampling frame from largest to smallest enrollment and assigned a continuous sequence of unique numeric identifiers. To maximize survey efficiency in line with GYTS methodology, we excluded schools with an enrolled eligible population of 40 or less. The cumulative population of eligible students in each school was then calculated.

To determine the sampling interval, we divided the total number of eligible students by 25, the target number of schools. A number-designated random start was then chosen randomly between 1 and the sampling interval. We first selected the school in which the cumulative population corresponding with the random start was located, after which subsequent schools were selected by adding sampling interval to random start until 25 schools were reached.

In the second stage, we selected classes by using systematic random sampling from a sequentially numbered list containing every eligible class in a selected school. Our calculated sampling interval for classes was based on the target student sample obtained from the enrollment list and assuming a fixed class size of 30. We specified the classes selected beforehand by their unique identifier. We surveyed all students in the selected classes who were present on the day of survey administration.

### Data collection

We collected information about students’ sociodemographic characteristics, use of smoked and smokeless tobacco, tobacco use cessation, SHS exposure, access to cigarettes, antitobacco and protobacco message exposure, and attitudes and beliefs about using tobacco by using a self-administered, semistructured questionnaire adapted from the GYTS Core Questionnaire version 1.2 with optional questions (18). We pretested the study instrument in 2 nonstudy secondary schools in Ibadan on March 11, 2021, to test for clarity of questions. To increase the participation and availability of eligible students, we avoided the examination period and days close to holidays. A team of 10 trained research assistants led by a team supervisor collected data from March 30, 2021, through June 4, 2021.

### Variable definition and measurement

Categories of variables of interest were tobacco use, SHS exposure, antitobacco advertising, protobacco advertising, attitudes and beliefs, and sociodemographics and background variables (eg, smoking by parents, peers) (Table 1).

**Table 1:** Tobacco-Related Characteristics of Adolescents (N = 3,199), Study of Prevalence and Predictors of Tobacco Use Among Adolescents Aged 11 to 18 Years, Ibadan, Nigeria, March–June 2021

Variable	Description	Response
**Tobacco use**		
Current cigarette smoking	Cigarette smoking on one or more days in the past 30 days	Yes, no
Current use of other smoked tobacco	Use of smoked tobacco products other than cigarettes (eg, pipes, cigars, water pipes, shisha, bidis) during the past 30 days	Yes, no
Current smokeless tobacco use	Use of smokeless tobacco products (eg, snuff, chewing tobacco leaf, tobacco toothpaste or tooth powder) in the past 30 days	Yes, no
Current use of any tobacco type	Use of cigarettes or other smoked tobacco products or smokeless tobacco products in the past 30 days	Yes, no
Dual tobacco	Use of smoked tobacco or smokeless tobacco during the past 30 days	Yes, no
Ever use tobacco (cigarettes, other smoked tobacco, smoked tobacco, smokeless tobacco, or any tobacco type)	Experimentation with any of the aforementioned tobacco products in the past	Yes, no
**Exposure to secondhand smoke**		
At home	Exposure to smoking at home on 1 or more days in the preceding 7 days	Exposed, not exposed
At school	Saw someone smoke inside the school building or outside on school property during the past 30 days	Exposed, not exposed
All exposure outside the home	Exposure in enclosed public places or at outdoor public places or in public transport vehicle or at school	Exposed, not exposed
All exposure	Exposure in the home, in enclosed public places, at outdoor public places, in a public transport vehicle, or at school	Exposed, not exposed
**Awareness of antitobacco advertising**		
Antitobacco messages in the media	Saw or heard any antitobacco messages in the media (eg, television, radio, internet, billboards, posters, newspapers, magazines, movies) in the past 30 days	Yes, no
Antitobacco messages at sporting or community events	Saw or heard any antitobacco messages at sporting or other community events in the past 30 days	Yes, no, or did not attend
**Awareness of protobacco advertising**		
Tobacco marketing at points of sale	Saw any tobacco marketing at points of sale in the past 30 days	Yes, no, or did not visit
Tobacco use in television programs, videos, or movies	Saw someone using tobacco on television, in videos, or in movies in the past 30 days	Yes, no, or did not watch
Tobacco use on the internet	Saw any advertisements for tobacco products on the internet in the last 30 days	Yes, no, or did not use
Exposure to free tobacco promotion	Were ever offered a free tobacco product from a tobacco company representative	Yes, no
Ownership of an object with a tobacco brand logo	Owned objects like a lighter, T shirt, hat, or sunglasses with a tobacco brand logo on it	Yes, no
**Attitudes and beliefs about using tobacco**		
Smoking helps people feel more comfortable socially	—	More comfortable, less comfortable, no difference
Young people who smoke have more friends	—	More friends, fewer friends, no difference
Smoking makes young people more attractive	—	More attractive, less attractive, no difference
Perception of smoking harmfulness	—	Definitely harmful, definitely not/unsure
**Sociodemographic characteristics**		
Age group, y	—	11–12, 13–15, 16–18
Sex	—	Male, female
Local Government Area^a^	—	Ibadan North, Ibadan North East, Ibadan North West, Ibadan South East, Ibadan South West
School type	—	Public, Private
Class	—	JS2, JS3, SS1
Student residence	—	Day student, boarding student
**Other background variables**		
Parents’ smoking status	—	One or both parents, none, do not know
Closest friends’ smoking status	—	None of them, some of them, most or all of them
Classmates’ smoking status	—	None of them, some of them, most or all of them
Access to cigarettes near school		Yes, no, or do not know
Perceived ease of getting cigarettes	—	Very difficult, fairly difficult, don’t know, fairly easy, very easy
Class teaching on dangers of tobacco use	—	Yes, no, or do not know

Abbreviations: —, not applicable; JS, junior secondary school; SS, senior secondary school.

a The lowest administrative level of government.

### Statistical analysis

We calculated weights for each student to account for the complex survey design and differential nonresponse at school, class, and student levels (27). The final weight for each student was a product of the school selection weight, class selection weight, and overall nonresponse adjustment factor. The school selection weight was the inverse probability of selecting a school. The class selection weight was the inverse probability of selecting a class in a school. The overall nonresponse adjustment factor was the product of school, class, and student nonresponse adjustment factors.

We used SPSS Statistics 23 (IBM Corp) to perform data analysis. The data collected were checked for errors and missing data, cleaned, and entered into the analysis software. Recoding of variables was done where appropriate. We summarized categorical variables with prevalence estimates and 95% CIs. We used the Pearson χ^2^ test to assess bivariate associations between independent variables and current tobacco use. We modeled a binary logistic regression for predictors of current use of tobacco. The significance level for entering a variable into the model was set at 10% from bivariate analyses, in addition to variables found to be important in the literature. Level of significance from the logistic regression was set at *P* <.05.

### Ethical considerations

We obtained ethical approval to conduct this study from the Oyo State Ministry of Health Ethical Review Committee. Before approaching the selected schools, we obtained approval from the Oyo State Ministry of Education. We then obtained approval from the heads of selected schools. We obtained written informed assent from each respondent after a detailed explanation of the study’s objectives, procedures, risks, and benefits and before starting the interview. The data were anonymized by using unique identifiers. Teachers were absent during survey administration to ensure privacy and to minimize reporting bias.

## Results

### Participation rates and sociodemographic characteristics

A total of 3,199 students were surveyed from 23 selected schools. The school, class, student, and overall response rates were school, 92%; class, 100%; student, 93%; and overall, 85.6%. Mean age of all respondents was 14.1 years (SD, ±1.4 years), with ages ranging from 11 to 18 years. Although all participants were adolescents, most respondents (70.5%) were aged 13 to 15 years, with a slightly higher proportion of female students (54.4%) than male students (45.6%) (Table 2).

**Table 2:** Sociodemographic Characteristics of Respondents, Study of Prevalence and Predictors of Tobacco Use Among Adolescents Aged 11–18 Years in Ibadan, Nigeria, March–June 2021 (N = 3,199)

Variable	Weighted percentages, % (95% CI)	Unweighted number
Age group, y		
11–12	13.2 (10.7–16.2)	419
13–15	70.5 (66.7–74.0)	2,251
16–18	16.3 (13.0–20.3)	529
Sex		
Male	45.6 (28.2–64.1)	1,466
Female	54.4 (35.9–71.8)	1,733
Local Government Area^a^		
Ibadan North	20.9 (11.3–35.3)	682
Ibadan North East	22.4 (10.1–42.7)	699
Ibadan North West	15.2 (6.2–32.9)	505
Ibadan South East	24.6 (10.9–46.4)	768
Ibadan South West	16.9 (8.6–30.5)	545
School type		
Public	91.8 (82.2–96.4)	2,969
Private	8.2 (3.6–17.8)	230
Class		
Junior secondary school 2	36.2 (31.4–41.3)	1,160
Junior secondary school 3	33.3 (32.4–34.2)	1,050
Senior secondary school 1	30.5 (26.1–35.3)	989
Student residence		
Day student	99.7 (98.2–99.9)	3,189
Boarding student	0.3 (0.1–1.8)	10

a The lowest administrative level of government.

### Prevalence and patterns of tobacco use

The prevalence of both ever and current use of all tobacco types was less than 5%. However, for all types, prevalence was higher in boys than girls. A very small proportion of respondents, 0.8%, were engaged in dual use of smoked and smokeless tobacco (Table 3).

**Table 3:** Prevalence and Patterns of Tobacco Use, Study of Prevalence and Predictors of Tobacco Use among Adolescents Aged 11–18 Years (N = 3,199) in Ibadan, Nigeria, March–June 2021

Variable	Weighted percentage (95% CI)
**Cigarettes**	
Ever use	
Male	3.1 (2.0–4.8)
Female	1.1 (0.5–2.1)
Overall	2.0 (1.3–3.0)
Current use	
Male	2.2 (1.3–3.9)
Female	0.6 (0.3–1.2)
Overall	1.4 (0.8–2.3)
**Other smoked tobacco**	
Ever use	
Male	2.3 (1.3–4.1)
Female	0.6 (0.2–1.8)
Overall	1.4 (0.8–2.4)
Current use	
Male	2.1 (1.1–3.9)
Female	0.5 (0.1–1.7)
Overall	1.2 (0.7–2.2)
**Smokeless tobacco**	
Ever use	
Male	2.2 (1.2–3.8)
Female	0.4 (0.1–1.1)
Overall	1.2 (0.7–2.0)
Current use	
Male	2.0 (1.1–3.7)
Female	0.2 (0.0–1.2)
Overall	1.1 (0.6–1.9)
**Any tobacco**	
Ever use	
Male	4.2 (2.7–6.5)
Female	1.3 (0.7–2.3)
Overall	2.6 (1.7–3.9)
Current use	
Male	3.6 (2.1–6.1)
Female	0.8 (0.4–1.7)
Overall	2.0 (1.3–3.3)
**Dual tobacco use (smoked and smokeless)**	
Male	1.4 (0.6–3.1)
Female	0.2 (0.0–1.2)
Overall	0.8 (0.4–1.7)

### Other tobacco use–related characteristics

Most respondents (97.6%) indicated that none of their parents smoked or that they were not aware of their parents’ smoking status. Similarly, most respondents indicated that none of their closest friends (93.4%) or classmates (89.1%) smoked tobacco. Few respondents (5.0%) had access to cigarettes near the school or perceived that cigarettes were easy to access (4.6%). Most (68.3%) perceived smoking to be harmful. A high proportion (40.3%) believed smokers had more friends (Table 4).

**Table 4:** Factors Affecting Tobacco Use, Exposure to Tobacco Advertising, and Exposure to Secondhand Smoke, Study of Prevalence and Predictors of Tobacco Use Among Adolescents Aged 11–18 Years (N = 3,199), Ibadan, Nigeria, March–June 2021

Variable	Weighted percentage (95% CI)
**Social influence**	
Parents’ smoking status	
One or both parents	2.4 (1.5–3.7)
None or don’t know	97.6 (96.3–98.5)
Closest friends’ smoking status	
None of them	93.4 (90.4–95.5)
Some of them	4.1 (2.8–6.0)
Most or all of them	2.5 (1.6–4.0)
Classmates’ smoking status	
None of them	89.1 (84.9–92.3)
Some of them	8.1 (6.0–10.9)
Most or all of them	2.8 (1.7–4.6)
**Access to cigarettes**	
Access to cigarettes near school	
Yes	5.0 (3.9–6.4)
None or don’t know	95.0 (93.6–96.1)
Ease of getting cigarettes	
Very difficult, fairly difficult, or do not know	95.4 (93.9–96.5)
Fairly easy or very easy	4.6 (3.5–6.1)
**School curriculum on tobacco**	
Class teaching on dangers of tobacco use	
Yes	55.9 (49.6–62.0)
None or do not know	44.1 (38.0–50.4)
**Attitude and perception to tobacco use**	
How smoking helps people feel socially	
More comfortable	19.0 (15.4–23.2)
Less comfortable	56.0 (48.9–62.9)
No difference	25.0 (20.0–30.9)
Young people who smoke have more friends	
More friends	40.3 (33.5–47.5)
Fewer friends	47.1 (41.8–52.5)
No difference	12.6 (9.4–16.6)
Smoking makes young people more attractive	
More attractive	10.1 (8.0–12.8)
Less attractive	73.6 (68.6–78.1)
No difference	16.3 (13.4–19.7)
Perception of smoking harmfulness	
Definitely harmful	68.3 (63.9–72.5)
Definitely not or unsure	31.7 (27.5–36.1)
**Antitobacco advertising**	
Awareness of antitobacco messages in the media	
Yes	43.3 (37.4–49.4)
No	56.7 (50.6–62.6)
Awareness of antitobacco messages at sporting or community events	
Yes	31.9 (27.0–37.2)
No or did not attend	68.1 (62.8–73.0)
**Protobacco advertising**	
Awareness of tobacco marketing at points of sale	
Yes	12.5 (10.4–15.0)
No or did not visit	87.5 (85.0–89.6)
Awareness of tobacco use on television, videos, or movies	
Yes	51.3 (47.4–55.3)
No or did not watch	48.7 (44.7–52.6)
Awareness of tobacco use on the internet	
Yes	9.8 (8.7–11.1)
No or did not use	90.2 (88.9–91.3)
Exposure to free tobacco promotion	
Yes	3.3 (2.2–5.0)
No	96.7 (95.0–97.8)
Ownership of an object with a tobacco brand logo	
Yes	6.9 (5.3–8.8)
No	93.1 (91.2–94.7)
**Exposure to secondhand smoke**	
Exposure to secondhand smoke at home	
Exposed	8.3 (6.8–10.0)
Not exposed	91.7 (90.0–93.2)
Exposure to secondhand smoke at school	
Exposed	12.0 (8.9–16.0)
Not exposed	88.0 (84.0–91.1)
All exposure to secondhand smoke outside the home	
Exposed	45.3 (41.3–49.2)
Not exposed	54.7 (50.8–58.7)
All exposure to secondhand smoke	
Exposed	46.1 (42.3–49.9)
Not exposed	53.9 (50.1–57.7)

Fewer than half of respondents were aware of antitobacco messages in the media (43.3%) and at sporting or community events (31.9%). About 51.0% of students were exposed to tobacco use through television, videos, or movies. Much lower proportions were exposed to tobacco use or marketing at points of sale (12.5%) or on the internet (9.8%). The lowest level of exposure to SHS was at home (8.3%), and 46.1% of respondents overall were exposed (Table 4).

### Predictors of current tobacco use

Boys were 3 times (aOR = 3.13; 95% CI, 1.53–6.42) more likely to use tobacco than girls (Table 5). Respondents, some of whose closest friends were smokers, were 3 times (aOR = 3.10; 95% CI, 1.77–5.41) more likely to use tobacco than those with no close friends who smoked. Those with access to cigarettes near their schools were twice (aOR = 1.97; 95% CI, 1.02–3.82) as likely to use tobacco as others, and those who perceived that cigarettes were easy to access were 7 times (aOR = 6.65; 95% CI, 2.55–17.33) likelier to use tobacco than other students. Additionally, those that felt adolescent smokers were attractive were 3 times likelier (aOR = 3.15; 95% CI, 1.17–8.44) to use tobacco than those who felt it was less attractive to smoke. Respondents who were not exposed to antitobacco messages at sporting or community events were less likely (aOR = 0.55; 95% CI, 0.39–0.76) to use tobacco than those who heard these messages. Finally, those who were aware of tobacco use on the internet were 3 times (aOR = 3.22; 95% CI, 1.48–7.04) likelier to use tobacco than other students. 

**Table 5:** Adjusted Logistic Regression Analysis of Association Between Selected Characteristics and Current Use of Any Tobacco Type, Study of Prevalence and Predictors of Tobacco Use Among Adolescents Aged 11–18 Years (N = 3,199) in Ibadan, Nigeria, March–June 2021

Variable	aOR (95% CI)	*P* value^a^
Age group, y		
16–18	2.00 (0.78–5.12)	.14
13–15	1.80 (0.47–6.94)	.36
11–12	1 [Reference]	—
Sex		
Male	3.13 (1.53–6.42)	.005
Female	1 [Reference]	—
Parents’ smoking status		
One or both parents	0.86 (0.38–1.95)	.67
None or don’t know	1 [Reference]	—
Closest friends’ smoking status		
Most or all of them	1.54 (0.53–4.45)	.39
Some of them	3.10 (1.77–5.41)	.001
None of them	1 [Reference]	—
Classmates’ smoking status		
Most or all of them	3.12 (1.15–8.49)	.03
Some of them	1.83 (0.87–3.85)	.102
None of them	1 [Reference]	—
Access to cigarettes near school		
Yes	1.97 (1.02–3.82)	.05
No or do not know	1 [Reference]	—
Ease of getting cigarettes		
Fairly easy/very easy	6.65 (2.55–17.33)	.001
Very difficult/fairly difficult/don’t know	1 [Reference]	—
Smoking makes young people more attractive		
More attractive	3.15 (1.17–8.44)	.03
No difference from non-smokers	1.98 (0.48–8.22)	.32
Less attractive	1 [Reference]	—
Perception of smoking harmfulness		
Definitely not or unsure	0.67 (0.42–1.06)	.08
Definitely yes	1 [Reference]	—
All exposure to secondhand smoke		
Exposed	2.93 (1.07–8.03)	.04
Not exposed	1 [Reference]	—
Awareness of antitobacco messages at sporting or community events		
No or did not attend	0.55 (0.39–0.76)	.002
Yes	1 [Reference]	—
Awareness of tobacco use on the internet		
Yes	3.22 (1.48–7.04)	.007
No or did not use	1 [Reference]	—

Abbreviations: —, not applicable; aOR, adjusted odds ratio.

a *P* value calculated by adjusted logistic regression; significant at *P* <.05.

## Discussion

The prevalence of tobacco use among adolescents in our study was low compared with recent global and African averages (10). It was also lower than findings from previous GYTSs across selected cities in Nigeria, including Ibadan (23), and another study done in Enugu, Nigeria (12). The low levels relative to global averages are likely due to the disproportionate burdens borne by Southeast Asia for smokeless tobacco and by the Americas and Europe for cigarettes (10). Similarly, the difference from the African average could reflect other countries in the region with higher prevalence (28). The difference as compared with the Enugu study is consistent with previous national findings showing higher prevalence of tobacco use in the southeastern part of Nigeria, where Enugu is located (29). These are all likely due to varying cultural and social norms.

The noted difference in tobacco use in Ibadan between the last round of GYTS in 2008 and our study may reflect tobacco control efforts in the intervening period. In the preceding decade, Nigeria signed on to and ratified the WHO FCTC and enacted the National Tobacco Control Act (22).

Levels of awareness of antitobacco messages in the media were lower than recent findings from different settings (28,30) as well as from the 2008 GYTS round in select Nigerian cities (23). It has been shown that sustained, well-designed, mass media campaigns can reduce tobacco use (31). It is therefore imperative that these campaigns form an important part of any comprehensive tobacco control program (31). However, global monitoring efforts have shown that low-income countries, including Nigeria, have not mounted an antitobacco media campaign in recent years (31).

Across all domains of protobacco advertising, the levels of exposure in our study were consistently lower than findings in most other studies (28,30). It is well documented that tobacco advertising, promotion, and sponsorship increase tobacco use and that comprehensive bans decrease tobacco use (32). Recent global monitoring reports recognize Nigeria as one of the best performing countries in adopting comprehensive advertising bans (31). The National Tobacco Control Act of 2015 (22) is exhaustive in articulating a ban on protobacco advertising, and country-specific monitoring has shown a high performance in this metric (20).

Despite the high performance of Nigeria with regards to bans on tobacco advertising, promotion, and sponsorship, more than half of respondents in our study were exposed to tobacco advertising through television, videos, or movies, suggesting a significant loophole in implementing the ban on these media. Additionally, awareness of tobacco use on the internet is noteworthy in our study. Despite the likely cross-border challenges that may be involved, policy makers are recognizing the need to ensure implementation of a ban on tobacco advertising, promotion, and sponsorship that use this channel of communication, considering the extensive exposure of children and adolescents to the internet’s various platforms (31).

Levels of exposure to SHS at home among our respondents were much lower than recent findings from countries across different continents (28,30), except for Finland where the values were slightly lower (33). Similar patterns were also found with levels of exposure in school. The 2 key factors that determine prevalence of SHS exposure are background prevalence of smoking in the population and variations in smoke-free laws and their enforcement (34). The low prevalence of SHS exposure in low-income settings such as Nigeria is mostly attributable to low smoking prevalence (34).

The effect on smoking of having close friends who smoke has also been shown in other studies (35–37). Adolescents are known to have strong social ties to friends (38), thus making them susceptible to peer pressure. The effect of peer pressure also explains the effect of having classmates who smoke. Similarly, perceived ease of getting cigarettes (37) supports the habit of smoking tobacco. Adolescents are exploratory and thrill-seeking in nature, and easy access to cigarettes only serves to feed these attributes. However, an added dimension is the effect on current tobacco use of access to cigarettes near school. This may play a role in the continued recruitment of children and young adolescents as lifelong tobacco users. Previous findings have shown that to introduce tobacco use to young people, tobacco companies strategically place youth-oriented brands in locations where young people congregate (39).

Our finding that the perception that smoking makes young people more attractive aligns with what was found in a Nigerian study conducted in 2013 (35). That study found that a positive attitude toward smoking was a significant determinant of smoking initiation among adolescent students. We also found that any exposure to SHS was a determinant of current tobacco use, as found in other studies (37,40). Witnessing tobacco use by peers, teachers, parents, siblings, or other adults sets an example for these very impressionable adolescents and normalizes tobacco use as something socially acceptable.

Finally, the effect of exposure to tobacco use on the internet is noteworthy because adolescents and children increasingly spend time on the internet and engage with different social media platforms. Highly engaging marketing and media advertising are effective at promoting tobacco use (41). Content on most internet and social media platforms is engaging and interactive.

Our study used a large sample size, which ensured the validity of various analyses and subgroup analyses. Respondents were selected by using standardized, systematic, and rigorous methods that provided representation of the in-school adolescent population in the study location. The approach of using application of weights and complex samples was an added strength. Both factors ensured generalizability and comparability to previous similar studies (12,28).

Our study had some limitations. First, the cross-sectional study design made it impossible to establish causality between independent and dependent variables through the demonstration of the time-sequence criterion. Second, all data were based on self-reports, possibly leading to under- or overreporting of behaviors, experiences, and perceptions and to recall bias. Although the extent of this under- or overreporting of behavior could not be determined, some GYTS questions have been analyzed and have demonstrated good test–retest reliability and validity (42). Third, this was a school-based design and was limited to students. It may, therefore, not be representative of all adolescents in Ibadan. However, in many countries, most people in the age group studied attend public, private, or technical schools. This holds true for our study area given that the net attendance ratio for Oyo state is about 70% (25), a value likely to be higher for Ibadan, the major urban area and capital city. We found a differential nonresponse rate between public and private schools at the school level, 0% and 33%, respectively. Although this may have altered the sample representativeness, we believe that this effect was minimal because the population of public-school students normally far outweighs that of private schools.

Our study provides information about prevalence of tobacco use and associated factors among in-school adolescents in Ibadan, Nigeria, and confirms that tobacco use was low but was associated with peer influence, access to cigarettes, tobacco use misperceptions, exposure to SHS, and protobacco advertising. Policy makers need to consider implementing an antitobacco campaign that uses a peer education strategy among adolescents along with enforcement of comprehensive bans on protobacco advertising and public smoking. A more comprehensive nationwide survey and maintenance of continuous surveillance is needed.
